# Preoperative Assessment of Medication-Related Osteonecrosis of the Jaw Using [18F]fluoride Positron Emission Tomography (PET)/CT and [18F]fluorodeoxyglucose PET/MRI in Correlation with Histomorphometry and Micro-CT—A Prospective Comparative Study

**DOI:** 10.3390/diagnostics14040428

**Published:** 2024-02-15

**Authors:** Christian Philipp Reinert, Christina Pfannenberg, Brigitte Gückel, Helmut Dittmann, Christian la Fougère, Konstantin Nikolaou, Siegmar Reinert, Rouven Schönhof, Sebastian Hoefert

**Affiliations:** 1 Department of Radiology, Diagnostic and Interventional Radiology, University Hospital Tübingen, 72076 Tübingen, Germany; christina.pfannenberg@med.uni-tuebingen.de (C.P.); brigitte.gueckel@med.uni-tuebingen.de (B.G.); konstantin.nikolaou@med.uni-tuebingen.de (K.N.); 2 Department of Radiology, Nuclear Medicine and Clinical Molecular Imaging, University Hospital Tübingen, 72076 Tübingen, Germany; helmut.dittmann@med.uni-tuebingen.de (H.D.); christian.lafougere@med.uni-tuebingen.de (C.l.F.); 3 Cluster of Excellence iFIT (EXC 2180) “Image Guided and Functionally Instructed Tumor, Therapies”, University of Tübingen, 72076 Tübingen, Germany; 4 German Cancer Consortium (DKTK), Partner Site Tübingen, 72076 Tübingen, Germany; 5 Department of Oral and Maxillofacial Surgery, University Hospital Tübingen, 72076 Tübingen, Germany; siegmar.reinert@med.uni-tuebingen.de (S.R.); rouven.schoenhof@med.uni-tuebingen.de (R.S.); sebastian.hoefert@med.uni-tuebingen.de (S.H.)

**Keywords:** fluorodeoxyglucose F18, fluorides, positron emission tomography, osteonecrosis, diphosphonates

## Abstract

Objectives: The purpose of this study was to investigate the imaging characteristics of medication-related osteonecrosis of the jaw (MRONJ) using [18F]fluoride positron emission tomography/computed tomography (PET/CT) and [18F]fluorodeoxyglucose (FDG) PET/magnetic resonance imaging (MRI) for preoperative assessment and to correlate them with microarchitectural and histomorphometric data with respect to clinical findings. Methods: Twelve patients (five female; mean age 75 ± 7.6 yr) with symptomatic MRONJ underwent both scans on the same day, and imaging findings were used to plan surgical interventions for seven patients. Bone tracer uptake was classified as high, medium, or low, and surgical samples were evaluated using Micro-CT and histomorphometric analysis. Results: CT showed medullary sclerosis in all patients, and MRI revealed gadolinium enhancement in four patients. PET imaging revealed remarkably elevated [18F]fluoride uptake and moderately increased [18F]FDG uptake in MRONJ compared to healthy jawbones, with both differences being statistically significant. [18F]fluoride uptake was associated with necrosis, bacteria, and inflammatory tissue. Micro-CT data did not show significant differences, but histomorphometric analysis revealed higher osteocyte and lacunae densities in the high [18F]fluoride uptake group, and more necrotic bone in the medium [18F]fluoride uptake group. Bacteria were observed in all areas. Conclusions: In summary, [18F]fluoride PET accurately identified MRONJ extent, revealing functional changes in jawbone remodeling not visible on CT. [18F]FDG PET showed differences in bone and soft tissue, though less pronounced. This method aids in evaluating disease activity and guiding treatment planning, requiring further research for optimal surgical approaches based on tracer uptake.

## 1. Introduction

Medication-related osteonecrosis of the jaw (MRONJ) is a severe adverse effect of antiresorptive therapy that significantly impacts the quality of life of affected patients. Bisphosphonates (BPs) and denosumab (DNO), a monoclonal antibody against the receptor activator of nuclear factor kappa-Β-ligand (RANKL), inhibit osteoclastic bone resorption [1,2,3].

Prolonged use and high doses of antiresorptive drugs raise MRONJ risk [4], defined as exposed jawbone or palpable bone via intraoral or extraoral fistula for over 8 weeks without jaw radiation history or metastatic disease [5]. The specific cause of MRONJ remains unclear, with hypotheses like inhibited osteoclast differentiation and decreased bone resorption [6]. Antiresorptive agents’ site-specific effects on bone metabolism are understood, but why MRONJ occurs in the jawbone is not entirely clear.

In recent years, the prevalence of MRONJ has significantly risen due to antiresorptive drug usage in various clinical applications [7]. The American Association of Oral and Maxillofacial Surgeons (AAOMS) proposed a four-stage classification system based on the extent and degree of bone infection [8]. Stages 1 and 2 are usually managed conservatively, although cure rates may be lower than those with extensive surgery [1,2,3,9]. The German Guideline recommends early surgery for early stages [10]. The diagnosis stage of MRONJ is prognostic and affects treatment success, with a reduced likelihood of cure in advanced stages. Early detection before progression to higher stages is therefore increasingly critical [11]. 

The standard preoperative assessment of patients with MRONJ involves a clinical examination of the jaw, along with panoramic radiography (orthopantomography), and, more recently, cone-beam computed tomography (CBCT), which provides a comprehensive three-dimensional morphological analysis of the jawbone to gather initial information on bone structure, extent, course, and disease progression [3,12]. In advanced stages or before surgical intervention, the use of multidetector CT (MDCT), magnetic resonance imaging (MRI), or bone scintigraphy is beneficial in determining the disease extent, establishing treatment options, and monitoring the response to therapy [13]. Of these imaging techniques, single-photon emission computed tomography (SPECT) shows promise for the early detection and monitoring of MRONJ activity in affected bone [14].

For successful treatment planning, precise determination of bony margins is crucial. CT and MRI have high diagnostic accuracy in detecting MRONJ, but limited ability to assess disease extent [15]. Studies show 99mTc bone scintigraphy and [18F]FDG PET/CT detect early MRONJ-induced bone metabolism changes [16,17]. However, bone scintigraphy provides limited anatomical information, and both methods have lower sensitivities than [18F]fluoride bone scanning [18,19].

MRONJ diagnosis is challenging due to nonspecific clinical and imaging findings, particularly when exposed bone is not evident [20]. Radiologic signs are scarce, and correlation with clinical signs may not always be present [21]. [18F]fluoride PET/CT is a promising technique for detecting subtle increased bone remodeling [22], but more research is needed to understand its pathoetiological significance.

The objective of our study was to examine the morphological and metabolic imaging characteristics of MRONJ using [18F]fluoride PET/CT and [18F]FDG PET/MRI in patients prior to surgery, and to establish a correlation with Micro-CT and histomorphometric data in order to assess clinical outcomes in surgically treated patients.

## 2. Materials and Methods

### 2.1. Patient Cohort

The local ethics committee approved and reviewed this prospective descriptive study (Project number: 536/2014BO1), and all enrolled patients provided informed consent for their data to be used for research purposes. The patient cohort consisted of twelve individuals (five female) with a mean age of 75 ± 7.6 years, who were admitted to our institution between February 2018 and October 2020 for pretherapeutic evaluation. All patients exhibited non-specific clinical symptoms that required an accurate diagnosis to identify the location of MRONJ manifestation for preoperative planning. MRONJ was diagnosed based on exposed bone in accordance with the AAOMS diagnostic criteria [23]. To prevent the distortion of tracer uptake analysis, patients with bone metastasis in the jawbone were excluded from the study.

### 2.2. Study Protocol

All patients underwent both [18F]FDG PET/MRI and [18F]fluoride PET/CT examinations on the same day, with the latter scheduled five hours after the initial examination to ensure sufficient time for tracer clearance. Due to the physical half-life of [18F]FDG, which is 110 min [24], the concentration of [18F]FDG had declined to 10% at the time of the [18F]fluoride PET/CT examination. Prior to the intravenous injection of [18F]FDG, patients underwent a 12 h fast. After completion of the [18F]FDG PET/MRI, there was no requirement for patients to prolong fasting.

### 2.3. [18F]FDG PET/MRI

The tracer dose was adjusted according to body weight (average: 84.8 ± 28.7 MBq). PET/MRI scans were performed using a Biograph mMR^®^ scanner (Siemens Healthineers, Erlangen, Germany). PET imaging started 40 min after injection, covering the area from the skull base to the clavicle in a single bed position, with a 30 min acquisition time. PET data were reconstructed using a 3D algorithm with specific parameters (matrix: 256 × 256, Gaussian filter: 4 mm). For the generation of a segmentation-based PET attenuation correction map, a 3D T1-weighted spoiled gradient-echo sequence with Dixon-based fat–water separation was acquired. The MRI protocol included a T2-weighted transversal and coronal turbo spin echo (TSE) sequence, T2-weighted and T1-weighted fast spin echo isotropic 3D sequences allowing multiplanar reformats (sampling perfection with application-optimized contrasts using different flip angle evolution [SPACE^®^], Siemens Healthcare GmbH, Erlangen, Germany), diffusion weighted imaging (DWI), and T1-weighted volumetric interpolated breath-hold examination (VIBE) sequences with gadolinium-based MRI contrast media (GADOVIST^®^, Bayer Vital GmbH, Leverkusen, Germany). MRI contrast agent was intravenously injected at 0.1 mmol/kg, with an average dose of 7.5 ± 2.6 mL. 

### 2.4. [18F]fluoride PET/CT

Using a Biograph mCT scanner (Siemens Healthineers, Erlangen, Germany), [18F]fluoride PET/CT imaging started 60 min after the intravenous injection of weight-matched [18F]fluoride (average activity: 85 ± 29 Mbq). Patients were immobilized with a vacuum mattress to minimize motion and artifacts. CT scanning had no contrast agent, using a 0.6 mm slice thickness and bone image reconstruction kernel. PET data were acquired from the skull base to the clavicle in a two-minute acquisition using a 3D ordered subset expectation maximization algorithm (two iterations, 21 subsets, Gaussian filter 2.0 mm, matrix size 400 × 400, and slice thickness 2.0 mm).

### 2.5. Image Analysis

The image analysis was conducted by a consensus of two radiologists, one oral and maxillofacial surgeon, and one nuclear medicine specialist. Qualitative and quantitative analyses were performed on both affected and healthy jawbones, including PET tracer uptake, CT Hounsfield units (HUs), and MRI parameters.

Bone was categorized as “high uptake” when the radiotracer activity in the affected bone and the surrounding tissue exceeded three times the background radiotracer activity observed in healthy bone structures and soft tissue.

Regions with a radiotracer uptake lower than “high uptake” but still higher than the background were classified as “medium uptake”, whereas those with no uptake exceeding the background were classified as “no uptake”. CT images were evaluated for periosteal thickening, focal erosions, or medullary sclerosis.

For quantitative analysis, CT HUs of the jawbone were measured using two-dimensional regions of interest (ROIs) in each region of the mandible, carefully excluding the cortical bone and teeth. Mean attenuation (HU) values with standard deviation (SD) were recorded for each CT ROI. The mean HUs of MRONJ-affected jaws and healthy jaws were calculated separately, and a semiquantitative index was calculated by dividing the mean HUs of MRONJ-affected jaws by the mean HUs of healthy jaws.

PET standard acquisition values (SUVs) of the mandible were measured using 50% isocontour volumes of interest (VOIs) in each region of the mandible. For each PET VOI, the mean SUV was recorded with SD. The mean SUV of MRONJ-affected and healthy jawbones and the calculated semiquantitative index for both examinations were documented.

### 2.6. Bone Specimens

During the surgical procedure, bone samples were collected based on the preoperative tracer uptake and categorized as having no (sequestrum), medium, or high tracer uptake, as designated by markings and localization. In total, 27 bone specimens were analyzed.

### 2.7. Surgical Technique

The surgical technique employed in this study was in accordance with the methodology described elsewhere [25]. Furthermore, the extent of resection was determined based on the findings from [18F]fluoride PET/CT and [18F]FDG PET/MRI as follows: Non-vital necrotic bone and adjacent hypermetabolic bone were resected. Bordering bone was assessed for adequate signs of bleeding, smoothed to eliminate any sharp edges, and closed with dense soft tissue coverage, in concordance with the German AR-ONJ position paper [10]. Bleeding bone was presumed to be viable. A submandibular wound drain was inserted, which, before closure, was irrigated with a gentamicin solution (one ampoule of gentamicin per 10 mL of sodium chloride solution). Sutures were removed after 14 days, with tube feeding for three days post surgery and drain removal after three days. All surgical procedures were performed by a single surgeon. Antibiotics were initiated 7 to 14 days prior to surgery, administered intravenously for seven days (starting one day before surgery and continuing for five days post surgery), followed by an additional week of oral antibiotics. The choice of antibiotics was adjusted based on intraoperative microbiological swab results. Samples were preoperatively planned through mapping on prints and a 3D printer model, and these locations were marked on the model for later reference. Additional samples taken during surgery were also annotated on the model and printout.

### 2.8. Micro-CT and Histomorphometric Analysis

Bone samples were fixed in 4% formalin and scanned using a Siemens Inveon Micro-CT machine (80 kVp, 192° rotation, 15 µm slice thickness). Micro-CT data were processed with microCAT II Software and converted to DICOM format. For microarchitectural analysis, we utilized the MicroView-Software (Version: Standard 2.5.0-3768, Parallax Innovations, General Electric, London, ON, Canada). Each sample was assessed by defining the bone threshold within the software and transferring the value to the “Isosurface-Tool” for visualization and reconstruction, with a voxel size of 15 × 15 × 15 µm. Next, we defined a cuboid volume of interest (VOI) for the trabecular bone area and determined measurement parameters including bone volume fraction (BV/TV), bone surface density (BS/BV), trabecular thickness (Tr.Th.), trabecular number (Tr.N.), trabecular space (Tr.Sp.), Euler characteristic (connectivity), and bone mineral density (BMD). All variables were measured in relative units.

**Figure 1:**
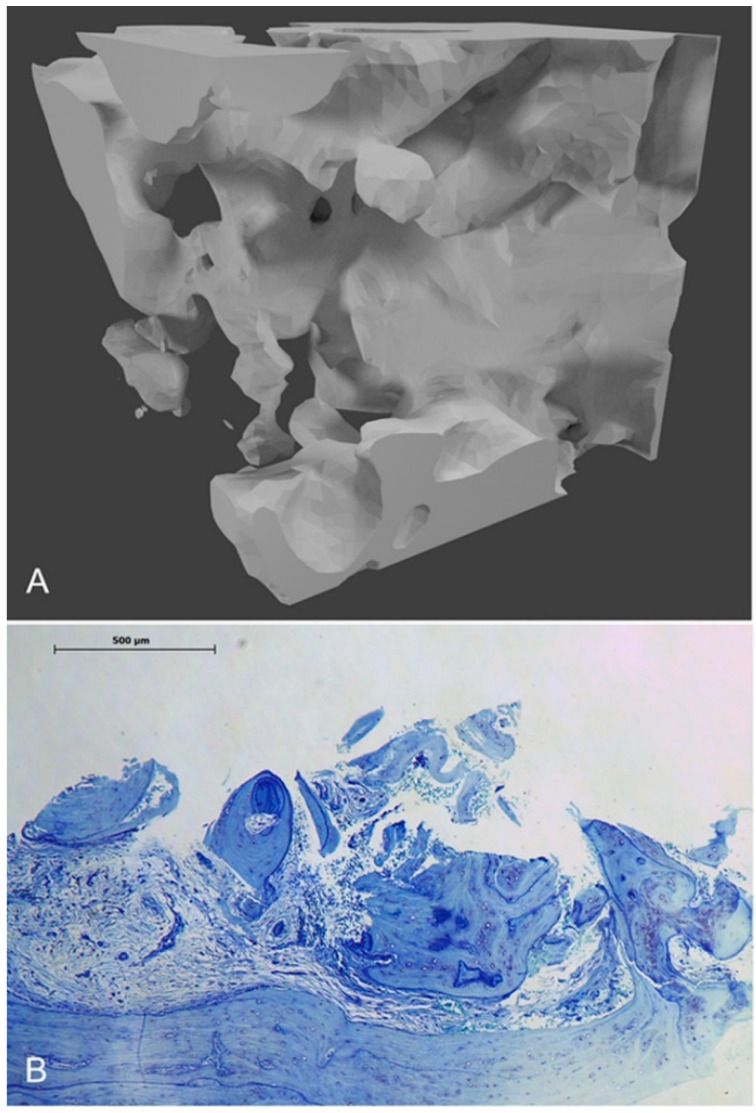
The Micro-CT and histological staining using toluidine blue are presented for a patient exhibiting elevated [18F]fluoride uptake. The spongiosa’s trabecular architecture is visible in the Micro-CT image (**A**), whereas the histological staining (**B**) displays granulation tissue, vital bone (osteocytes), and osteoblast lining, with an inlet scale bar, at 4× magnification.

After Micro-CT scanning, samples were decalcified, embedded, and microsectioned (3 µm). Stains, including H&E, Movat Pentachrom, Toluidin Blue, Elastica van Gieson, and Azan (Figure 1), followed the manufacturer’s instructions. ImageJ (NIH, Bethesda, MD, USA, Version 1.54h) analyzed five fields at 20× magnification for each stain, quantifying osteocyte and lacunae densities (osteocyte/mm^2^ and lacunae/mm^2^) per specimen. Osteoblast and osteoclast densities per bone surface were calculated. Resorption lacunae length by osteoclasts was measured. Tissue analysis used the Allred Score for infection, granulation, connection, bacteria presence, and necrotic bone. Micro-CT and histomorphometric analyses were blinded to patient information. 

### 2.9. Statistics

The statistical analysis was conducted using IBM SPSS version 22 for the radiological data and JMP version 16.2.0 from the SAS Institute for the histological data. The normality of all parameters was tested using the Kolmogorov–Smirnov test. To compare the differences in the Micro-CT, histomorphometry, PET parameters, CT, and MRI features between the affected and healthy jawbones, a Wilcoxon test was utilized. To address the multiple comparisons, a Benjamin Hochberg correction was applied. The adjusted *p*-values were considered significant at a level of 0.05. An Allred Intensity Score [26] was employed to categorize histological findings into groups based on the extent of histomorphometric findings, classifying them as either “low”, “moderate”, or “high”.

## 3. Results

### 3.1. Patient Characteristics

As of July 2022, patients were followed up for 21 to 53 months based on imaging. Seven out of twelve patients underwent surgical removal of the affected jawbone areas, while five chose a conservative approach after diagnosis. Surgical specimens from 31 samples were analyzed, including regions with “high uptake”, “medium uptake”, neo-bone, sequestrum, and healthy bone. 

All patients included were diagnosed with stage 2 disease in the lower jaw. These patients opted for surgical intervention and were included in the study for presurgical diagnostics and planning. This procedure involved the removal of necrotic bone (areas with no uptake) and the excision of adjacent bone exhibiting high uptake, followed by the smoothing of the surrounding bone. Subsequently, the wound was closed with soft tissue coverage, and antibiotic treatment was administered as per the methods described and detailed elsewhere [25]. For patients who chose not to undergo surgery following PET imaging, conservative treatment was pursued. Among the surgical patients, complete healing was observed post surgery, with the exception of one patient who developed a new necrotic lesion in a different location and jaw. Detailed information regarding surgically treated patients and their respective follow-up periods can be found in Table 1a. In contrast, conservative treatment (Table 1b) led to temporary improvement in the disease but did not result in complete healing, except in a single patient.

### 3.2. Qualitative Imaging

**Figure 2:**
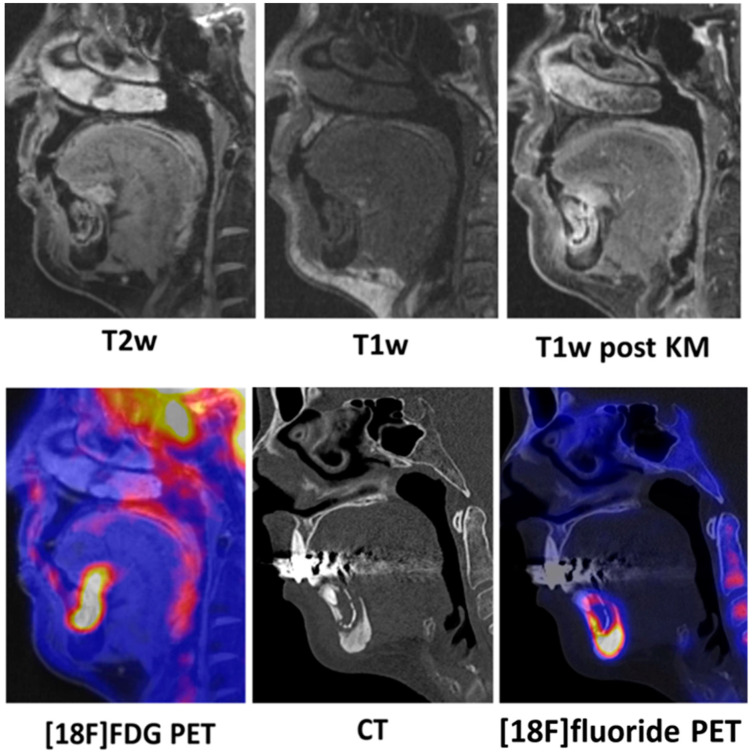
A 58-year-old female patient diagnosed with medication-related osteonecrosis of the jaw (MRONJ) in the mandible. Sequestrations are visible in regions 34 to 43. In the sagittally reconstructed images, the sequestration area within the jawbone appears hyperintense in T2-weighted MRI images and hypointense in T1-weighted images, indicating bone edema. In the post-contrast T1-weighted images, there is a notable enhancement of gadolinium in the affected mandible and, to a lesser extent, in the surrounding soft tissue. To a comparable extent, there is an uptake of [18F]fluorodeoxyglucose (FDG) in the sequestration area and adjacent soft tissue, consistent with inflammation-related granulation tissue. Moreover, no significant soft tissue inflammation was observed. On CT imaging, there is evidence of increased sclerosis in the mandible surrounding the sequestration area, accompanied by corresponding elevated [18F]fluoride uptake on positron emission tomography (PET), while the sequestration area itself shows no [18F]fluoride uptake. Overall, the extent of regions with increased metabolic activity on [18F]fluoride PET was smaller than the morphological changes detected by CT and MRI.

Trabecular sclerosis was observed in the affected jawbone of all 12 patients when compared to the healthy jawbone, as determined by CT. Additionally, sequestrations were present in five patients. MRI revealed increased gadolinium accumulation in four patients, but bone marrow edema was not detected. The periosteal soft tissue surrounding the affected jawbone showed increased gadolinium enhancement in all cases. PET imaging showed increased [18F]fluoride uptake in the affected jawbone compared to the healthy jawbone in 10 patients, although the extent of the affected regions with increased metabolic activity on [18F]fluoride PET was smaller than the morphologic changes observed by CT and MRI in all cases (Figure 2). The [18F]FDG PET revealed lower levels of tracer uptake in the affected jawbone regions compared to [18F]fluoride PET.

### 3.3. Quantitative Imaging 

The trabeculae of the affected jawbone had a higher mean HU compared to the healthy jawbone (1085 ± 218 HU vs. 271 ± 136 HU, SQI 6.5 ± 7.4, *p* < 0.001). On the other hand, the T1 signal was lower in the affected jawbone (140 ± 58) compared to the healthy jawbone (303 ± 124, SQI 0.5 ± 0.3, *p* < 0.001). However, there was no significant difference in the T2 signal between the two. In addition, the amount of gadolinium enhancement did not differ between the affected and healthy jawbones (304 ± 143 vs. 281 ± 151, SQI 1.4 ± 0.7, *p* > 0.05). The [18F]fluoride uptake was significantly higher in the affected jawbone than in the healthy jawbone (21 ± 12 vs. 2.4 ± 0.8, SQI 8.9 ± 3.3, *p* < 0.001). Meanwhile, the [18F]FDG uptake was moderately but significantly higher in the affected jawbone than in the healthy jawbone (1.1 ± 0.4 vs. 0.6 ± 0.2, SQI 2.0 ± 0.9, *p* < 0.01). This increase was also observed in the adjacent perimandibular soft tissue (4.1 ± 1.4 vs. 1.9 ± 0.3, *p* < 0.001). More detailed results can be found in Table 2.

### 3.4. Microarchitecture

There were no variations observed in the trabecular thickness (Th.Th.) and bone surface density (BS/BV) across all groups (Table 3). However, the high uptake group displayed higher BMD values compared to the other groups, which was also apparent in the BV/TV and trabecular number (Tr.N.). The Euler Index showed no differences among the groups. However, when it comes to trabecular space (Tr.Sp.), the high uptake-bone had the lowest value in contrast to the other groups. The differences noted in the various measurements between the groups were not statistically significant.

### 3.5. Histomorphometry

The osteocyte density differed significantly among the groups (Table 4). The bone with the highest [18F]fluoride uptake had the highest osteocyte density (335.5 cells/mm^2^), followed by the bone with medium hot uptake (164.4 cells/mm^2^), and the necrotic bone had the lowest osteocyte density, as expected, with the absence of osteocytes in the lacunae. However, a few cells were still visible. The bone with the highest [18F]fluoride uptake had the highest lacunae density (444.3 cells/mm^2^), and the difference from the bone with medium uptake (305.1 cells/mm^2^) was significant. The necrotic bone was in between but without a significant difference. Interestingly, the osteoblast density did not differ significantly among the groups and was between 24.5 and 42.9 cells/mm. Similar results were found for osteoclasts per surface length, which ranged from 1.5 to 1.6 cells/mm in all groups. The mean length of resorption lacunae was the shortest in the bone with the high [18F]fluoride uptake, followed by the two other groups. However, no significant differences were observed (Table 4). 

**Figure 3:**
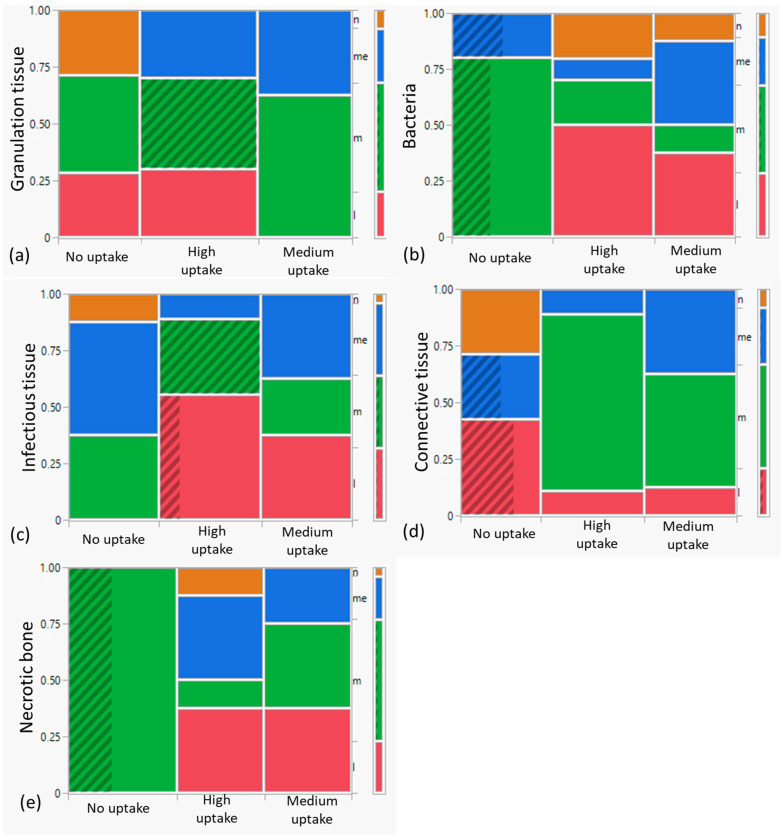
The Allred Intensity Score of granulation tissue (**a**), bacteria (**b**), infectious tissue (**c**), connective tissue (**d**), and necrotic bone (**e**) was assessed in samples categorized by tracer uptake (no uptake, medium uptake, and high [18F]fluoride uptake). Significant differences were observed among all the groups. n = none; me = medium; m = much; l = less.

The bone with medium [18F]fluoride uptake had the highest significant amount of granulation tissue compared to the bone with high [18F]fluoride uptake, and the bone without [18F]fluoride uptake showed the least amount of this tissue (Figure 3a). Bacteria and infectious tissue were most abundant in bone without [18F]fluoride uptake with similar amounts in the bone with medium and high [18F]fluoride uptake (Figure 3b,c). Conversely, the bone with the highest [18F]fluoride uptake had the most connective tissue, followed by the bone with medium [18F]fluoride uptake, and the difference was significant (Figure 3d). The bone without [18F]fluoride uptake had the highest amount of necrotic bone, followed by the bone with medium [18F]fluoride uptake, and the bone with the highest [18F]fluoride uptake had the least amount of necrotic bone (Figure 3e). 

**Figure 4:**
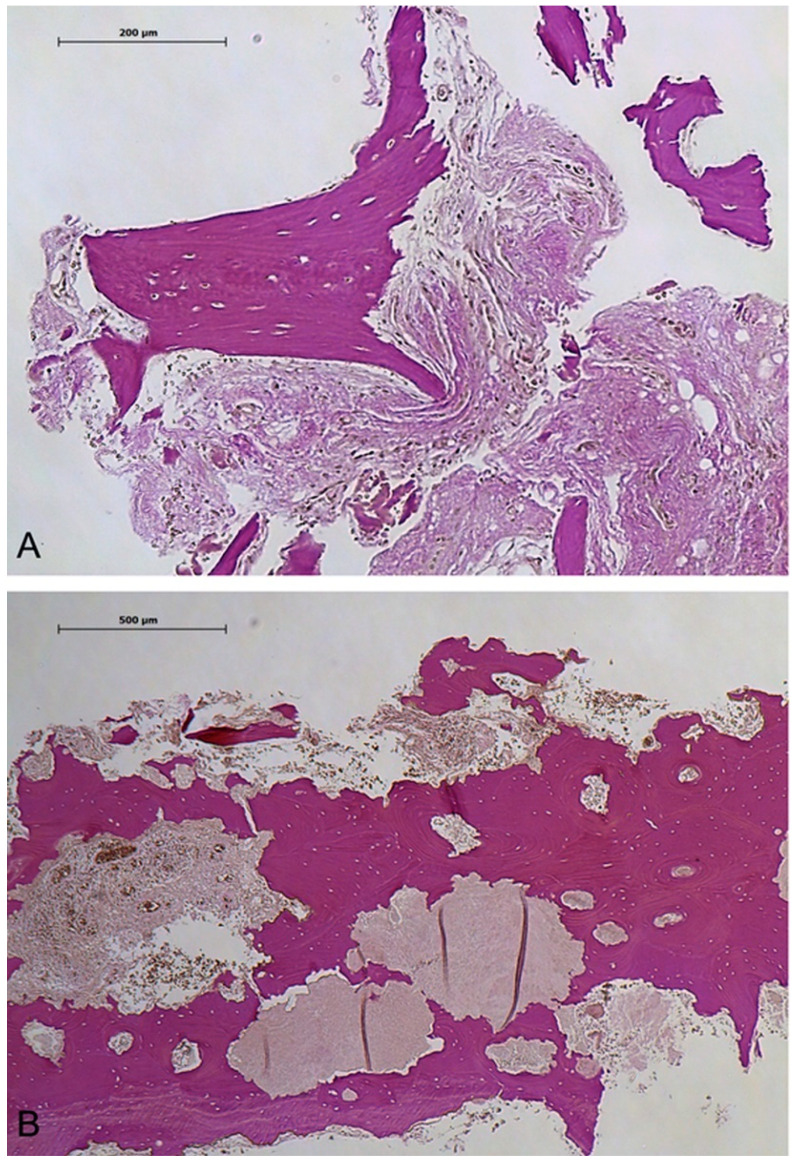
Samples of histology are presented with elastica van Gieson staining; (**A**) portrays a patient belonging to the high [18F]fluoride uptake group, displaying connective tissue and osteoblast lining at 10× magnification. (**B**) shows a patient from the group without [18F]fluoride uptake, depicting necrotic bone (with empty osteocyte lacunae), bacteria, and granulation tissue at 4× magnification, with an inlet scale bar.

Figure 4 shows histological samples stained with elastica van Gieson, providing visual evidence of histological differences between patients with high and low [18F]fluoride uptake (Figure 4).

## 4. Discussion

Managing MRONJ is challenging and involves either conservative or surgical approaches [1,9]. Both options have advantages and disadvantages in terms of sustained therapeutic efficacy and quality of life [1,2,3,9]. 

The current surgical approach involves removing bone that exhibits signs of necrosis and considering tracer uptake. However, it remains uncertain whether it is necessary to surgically remove bone with high or moderate uptake of [18F]fluoride and/or [18F]FDG. This study represents an initial effort to establish a connection between bone metabolism and histological, histomorphometric, and Micro-CT findings. Our findings provide initial evidence that stable surgical outcomes (cured) can be attained by surgically removing necrotic bone without tracer uptake and bordering bone exhibiting high tracer uptake.

We conducted diagnostic investigations using [18F]FDG PET/MRI and [18F]fluoride PET/CT to assess symptomatic MRONJ patients. Our goal was to analyze jawbone remodeling, including both morphological and metabolic changes, and correlate these findings with histomorphometric analysis. This study provides valuable insights into functional alterations in MRONJ’s bone structure that are not discernible with conventional MRI or CT imaging.

Our results revealed specific characteristics in jawbones affected by MRONJ, including trabecular sclerosis, periosteal reaction, and cortical destruction. We observed a significant increase in [18F]fluoride uptake, extending beyond the visibly affected areas of MRONJ on CT, indicating metabolic changes in the jawbone. Regions with sequestra were associated with periosteal neo-ossification, and [18F]fluoride uptake in these areas was below the background tracer activity level. These morphological changes in the jawbone structure associated with MRONJ are consistent with previous literature reports [20,27,28,29]. The periosteal bone surface is the primary site of jawbone remodeling, where formation modeling compensates biomechanically for the loss of bone marrow [30]. 

Our observations suggest that MRONJ is more common in the mandible due to its denser cortical bone and lower cancellous bone content compared to the mostly cancellous and well-vascularized maxilla [31]. Sclerosis is an early sign of MRONJ, characterized by trabecular structure changes like compaction and a “pumice-like” appearance [32]. The correlation between sclerosis severity and drug use duration remains unclear [33], and our studies suggest excessive bone growth with denosumab usage [34,35]. Advanced stages of MRONJ display medullary sclerosis with disorganized microtrabeculae and poor corticomedullary differentiation [27,36]. The literature emphasizes evaluating cortical bone, including bowl-shaped defects (sequestrum) and periosteal new bone formation (periosteal double contour), which appear later in MRONJ [37]. Our findings did not show microarchitectural differences in areas of tracer uptake, possibly due to the time required for such changes to develop [34].

Guggenberger et al. noted increased [18F]fluoride uptake in early bone remodeling changes before visible MRONJ [22,33,38]. Our study similarly observed higher [18F]fluoride uptake in morphologically affected jawbone compared to CT, suggesting the detection of subtle changes. However, osteoblast and osteoclast density remained unchanged in these areas. Medium-[18F]fluoride-uptake bone exhibited various tissue types, while high-uptake bone showed minimal bacterial presence, mostly in connective tissue. The absence of [18F]fluoride uptake indicated necrotic bone [22], with no correlation found between osteoblast numbers and high uptake areas, likely due to the abundance of connective tissue [39]. Necrotic areas in MRONJ show reduced bone metabolism, which was observed in bone scintigraphy scans [28]. However, scintigraphy lacks the specificity to differentiate tracer uptake within MRONJ from surrounding reactive bone [29]. [18F]fluoride PET/CT, with higher sensitivity and specificity in a shorter examination time, overcomes this limitation [40]. 

Histomorphometric analysis revealed higher osteocyte density in MRONJ-affected jawbones with high [18F]fluoride uptake. Intermediate tracer uptake areas had lower osteocyte density, while healthy jawbones showed the lowest density. Osteoblast and osteoclast density were higher in the affected jawbone, but not statistically significantly. Osteoblasts and osteoclasts form bone multicellular units (BMUs) at sites of bone turnover [41]. Osteoclasts break down the bone matrix formed by osteoblasts, affecting bone remodeling [42]. Antiresorptive medications like BP inhibit osteoclast function, leading to the prolonged presence of old bone, manifesting as periosteal neo-ossification and medullary sclerosis in the jawbone [42,43]. Osteoclast density did not differ among the groups, including necrotic bone, aligning with previous studies [39,44]. Patients received both denosumab and bisphosphonates, possibly explaining our results [44]. After intravenous administration, [18F]fluoride exchanges ions with hydroxyl groups in bone capillaries, forming fluoroapatite at sites of high bone turnover [10]. This may relate to our finding of high granulation tissue rates [45]. The study limitations may be influenced by anti-inflammatory therapy reducing tracer uptake [46]. Antibiotic use before surgery might minimally affect tracer uptake.

Our findings suggest varying granulation tissue extents depending on [18F]fluoride uptake, highest in the group with medium [18F]fluoride uptake. Bacterial and inflammatory processes contribute to MRONJ pathogenesis [47], but our results suggest limited impact on functional changes in jawbone remodeling. Bone with medium [18F]fluoride uptake may resemble necrotic bone more than high-[18F]fluoride-uptake bone, raising a surgical planning question: should bone with high [18F]fluoride uptake near necrotic bone be preserved for improved healing compared to bone with medium [18F]fluoride uptake near bone without [18F]fluoride uptake and sequestrum bone? Conservative treatment can stabilize the disease, relieve symptoms, and may be suitable for patients with poor general health or those declining surgery [9]. Surgical interventions should be carefully considered in terms of risk and benefit [48]. 

We acknowledge several limitations in our study. First, the size of our cohort was relatively small, highlighting the need for larger prospective studies that incorporate long-term outcome analyses. Second, utilizing intraoperative navigation could enhance the precision of bone sampling, albeit this was indirectly realized by utilizing 3D models. Future research should employ 3D navigation for more precise sample collection. An alternative intriguing option could have entailed pre-labeling the bone with tetracyclines for fluorescence-based identification and subsequently aligning the resection boundaries and resection site localization with fusion imaging and the categorization of examination groups [49]. However, we believe such an approach might introduce higher levels of inaccuracy. Our study is pioneering in its utilization of PET imaging to evaluate the extent of bone involvement in MRONJ in contrast to morphological characteristics, thereby eliminating the need for additional preoperative imaging. However, every clinical procedure for determining the bone artifact, especially in the border area of necrosis, is difficult and also has the consequence in our study of viewing the result critically and not absolutely. Further research is necessary to determine the specific bones that require surgical excision. 

Third, since all patients underwent [18F]fluoride PET/CT five hours after [18F]FDG PET/MRI, there remained 10% of [18F]FDG that might introduce bias in the quantification of [18F]fluoride uptake. However, considering the jawbone’s [18F]FDG uptake is not disproportionately high compared to the relatively increased [18F]fluoride uptake, we deem the impact of residual [18F]FDG uptake to be negligible in this context. Fourth, imaging was performed as part of the study protocol solely for therapy planning, and not all patients underwent surgical intervention. However, this reflects routine clinical practice, where treatment decisions are made based on a combination of imaging findings and clinical assessment. Fifth, although we conducted clinical observation, we did not obtain follow-up imaging data as part of the study, which would have allowed for comparative imaging analysis over time.

In summary, [18F]fluoride PET accurately identified MRONJ extent, revealing functional changes in jawbone remodeling not visible on CT. [18F]FDG PET showed differences in the bone and soft tissue, though less pronounced. This method aids in evaluating disease activity and guiding treatment planning, requiring further research for optimal surgical approaches based on tracer uptake. 

## Figures and Tables

**Table 1:** (**a**) Patient characteristics of surgically treated patients. All necroses were located in the lower jaw. (**b**) Patient characteristics of conservatively treated patients with local rinses, superficial debridement, and antibiotic treatment at inflammation surge.

**(a)**									
**Patient**	**Sex; Age**	**Primary Diagnosis**	**Antiresorptive Drug; Application**	**Duration of Treatment ^#^**	**Antibiotics**	**Duration of Antibiotic Treatment ^§^**	**Clinical Outcome after Surgery**	**Follow Up**	**Clinical Outcome at Endpoint of Follow Up**
1	male; 67 yr.	Prostate cancer	Zoledronate; iv	79 months	Clindamycin; Moxifloxacin	32 (25o; 7iv)	cured	30 months	cured
2	female; 76 yr.	Breast cancer	Zoledronate; iv	22 months	Cefuroxime; Moxifloxacin	32 (25o; 7iv)	cured	29 months	cured at origin; new necrosis at the upper jaw
3	female; 68 yr.	Breast cancer	Denosumab; sc	48 months	Sultamicillin; Ampicillin/Sulbactam; Metronidazole; Cefuroxime	29 (22o; 7iv)	cured	40 months	cured
4	male; 65 yr.	Kidney cancer	Denosumab; sc	6 months	Sultamicillin; Ampicillin/Sulbactam; Clindamycin	32 (25o; 7iv)	cured	18 months	cured
5	male; 65 yr.	Multiple myeloma	Zoledronate; iv	13 months	Sultamicillin; Metronidazole	40 (33o; 7iv)	cured	10 months	cured
6	male; 79 yr.	Multiple myeloma	Zoledronate; iv Denosumab; sc	24 months	Sultamicillin; Ampicillin/Sulbactam	22 (15o; 7iv)	cured	6 months	cured
7	male; 77 yr.	Multiple myeloma	Zoledronate; iv	7 months	Sultamicillin; Ampicillin/Sulbactam; Metronidazole	21 (14o; 7iv)	cured	5 months	cured
**(b)**									
**Patient**	**Sex; Age**	**Primary Diagnosis**	**Location of Necrosis**	**Antiresorptive Drug; Application**	**Duration of Treatment ^#^**	**Antibiotics**	Duration of Antibiotic Treatment *	**Follow Up**	**Clinical Outcome at Endpoint of Follow Up**
8	female; 53 yr.	Breast cancer	Lower jaw both sides	Denosumab (biannual)	18 months	none		60 months	spontaneous sequestrum; cured
9	female; 76 yr.	Breast cancer	Lower jaw both sides	Ibandronate Denosumab	138 months	Sultamicillin; Moxifloxacin	87 (80o; 7iv)	63 months	sequestrum; not cured
10	male; 68 yr.	Lung cancer	Lower jaw both sides	Denosumab	29 months	Metronidazole (local application)	each visit	7 months	sequestrum; not cured
11	male; 76 yr.	Prostate cancer	Lower jaw left	Zoledronate	18 months	Sultamicillin	280 (o)	12 months	died; not cured
12	female; 78 yr.	Breast cancer	Lower jaw both sides	Zoledronate Denosumab	58 months	Sultamicillin Sulfamethoxazole/Trimetoprima	263 (o)	40 months	not cured

^#^ Duration of therapy until onset of necrosis; ^§^ duration of antibiotic therapy at surgery (days); * duration of antibiotic therapy until last visit in total (days); intravenously: iv; subcutaneously: sc; orally: o; cured is defined as no signs of exposed bone, fistula, infection, or pain.

**Table 2:** Mean and standard deviation of quantitative imaging markers in both the affected jawbone and healthy jawbone.

Imaging Markers	Affected Bone	Healthy Bone	SQI	*p* Value
T2w	28 ± 19	40 ± 26	0.9 ± 0.4	>0.05
T1w	140 ± 58	303 ± 124	0.5 ± 0.3	0.0005
T1w post-contrast (bone)	304 ± 143	281 ± 151	1.4 ± 0.7	>0.05
[18F]FDG PET SUV_mean_ (bone)	1.1 ± 0.4	0.6 ± 0.2	2.0 ± 1.1	0.003
[18F]FDG PET SUV_mean_ (soft tissue)	4.1 ± 1.4	1.9 ± 0.3	2.2 ± 0.9	0.0004
[18F]fluoride PET SUV_mean_ (bone)	21 ± 12	2.4 ± 0.8	8.9 ± 3.3	0.00001
Hounsfield Units	1085 ± 218	271 ± 136	6.5 ± 7.4	0.00001

T2w = T2-weighted images; T1w = T1-weighted images; T1w post-contrast (bone) = T1-weighted post-gadolinium images; [18F]FDG PET = fluorodeoxyglucose positron emission tomography; SUV = standardized uptake value; SQI = semi-quantitative index.

**Table 3:** Micro-CT analysis was used to determine the mean, standard deviation, and range of necrotic bone areas, as well as bone areas with medium and high [18F]fluoride uptake. There were no significant differences in any of the values observed between the different groups categorized by tracer uptake.

Micro-CT	Necrotic Bone	Medium [18F]fluoride Uptake	High [18F]fluoride Uptake	*p* Value
BV/TV	0.24 ± 0.09	0.27 ± 0.14	0.33 ± 0.15	>0.05
	(0.12–0.49)	(0.14–0.52)	(0.14–0.6)	
BS/BV	4.41 ± 2.97	5.34 ± 2.05	5.42 ± 1.88	>0.05
	(2.3–10.48)	(2.78–8.61)	(2.78–8.61)	
Tr.Th. (mm)	0.48 ± 0.25	0.43 ± 0.17	0.41 ± 0.15	>0.05
	(0.19–0.87)	(0.25–0.71)	(0.23–0.72)	
Tr.N. (mm^−1^)	0.59 ± 0.10	0.63 ± 0.12	0.86 ± 0.10	>0.05
	(0.28–1.21)	(0.35–0.96)	(0.24–1.51)	
Tr.Sp. (mm)	1.62 ± 0.26	1.31 ± 0.31	1.14 ± 0.26	>0.05
	(0.54–2.75)	(0.65–3.64)	(0.65–2.26)	
Euler Index	1.36 ± 1.86	1.28 ± 0.87	2.13 ± 2.35	>0.05
	(0.19–5.69)	(0.31–2.97)	(0.13–8.10)	
BMD (g·cm^−3^)	656.0 ± 38.6	580.1 ± 46.1	750.0 ± 38.6	>0.05
	(567.2–764.5)	(555.5–997.2)	(519.5–937.9)	

BV/TV = bone volume fraction; BS/BV = bone surface density; Tr.Th. = trabecular thickness; Tr.N. = trabecular number; Tr.Sp. = trabecular space; BMD = bone mineral density.

**Table 4:** The bone samples were subjected to histomorphometric analyses to determine the density per area of osteocytes and bone lacunae, as well as the number of osteoclasts and osteoblasts per length of bone border in histological samples.

Histological Samples	Necrotic Bone	Medium [18F]fluoride Uptake	High [18F]fluoride Uptake	*p* Value
Osteocytes/mm^2^	10.2 ± 48.2	164.4 ± 53.6	335.5 ± 48.2	0.0005
Lacunae/mm^2^	347.5 ± 37.9	305.1 ± 42.3	444.3 ± 37.9	0.01
Osteoblasts/mm	24.5 ± 14.5	37.9 ± 8.9	42.9 ± 8.4	>0.05
Osteoclasts/mm	1.5 ± 0.5	1.6 ± 0.3	1.6 ± 0.3	>0.05
Resorption lacunae, mean length (mm)	0.033 ± 0.004	0.033 ± 0.004	0.025 ± 0.004	>0.05

## Data Availability

The data presented in this study are available on request from the corresponding author.
